# Immune checkpoint inhibitors in penile cancer

**DOI:** 10.2144/fsoa-2021-0044

**Published:** 2021-05-21

**Authors:** Carlo Buonerba, Luca Scafuri, Ferdinando Costabile, Bruno D’Ambrosio, Simona Gatani, Pasquale Verolino, Rossella Di Trolio, Vincenzo Cosimato, Antonio Verde, Giuseppe Di Lorenzo

**Affiliations:** 1 Centro di Referenza Nazionale per l'Analisi e Studio di Correlazione tra Ambiente, Animale e Uomo, Istituto Zooprofilattico Sperimentale del Mezzogiorno, Portici 80055, Italy; 2 Oncology Unit, Hospital ‘Andrea Tortora’, ASL Salerno, Pagani, Italy; 3 Multidisciplinary Department of Medical-Surgical & Dental Specialties, Plastic Surgery Unit, University of Campania Luigi Vanvitelli, Naples, Italy; 4 Unit of Melanoma, Cancer Immunotherapy & Development Therapeutics, Istituto Nazionale Tumori Istituto di Ricovero e Cura a Carattere Scientifico Fondazione G. Pascale, Naples, Italy; 5 Division of Laboratory Medicine, Civil Hospital ‘Maria SS. Addolorata’, ASL Salerno, Eboli, Italy; 6 Department of Medicine & Health Sciences ‘Vincenzo Tiberio’, University of Molise, Campobasso, Italy

**Keywords:** immune checkpoint inhibitors, immunotherapy, penile cancer

Penile cancer (PCa) is a rare yet deadly disease, with an incidence rate varying in the range of 1–10 cases per 100,000 men across countries worldwide and a 5-year survival rate ranging from 90% in stage I patients to a dismal 5% in stage IV patients [1]. With 300 men dying annually of advanced/recurrent PCa in the USA alone [2], effective systemic therapeutic options remain a highly unmet need, and only a few currently available chemotherapy agents are recommended on the basis of noncomparative prospective [3,4] or retrospective [5] studies. The EGFR may represent a valuable druggable target, based on the frequent expression of EGFR in PCa [6–8] and preliminary evidence of efficacy obtained with anti-EGFR agents [9], although more supporting prospective data are needed for approval and its widespread use in clinical practice.

The advent of immune check-point inhibitors (ICI), mainly directed at programmed death-1 (PD-1), programmed death ligand-1 (PDL-1) or cytotoxic t-lymphocyte antigen 4 (CTLA-4), has truly revolutionized therapy of solid malignancies and may also expand the limited treatment landscape of advanced PCa. In fact, PD-L1 expression, a known marker predictive of efficacy of anti-PD-1/PDL-1 inhibitors across different solid tumors [10], was reported in 51% of cases in a surgical cohort of 35 men with PCa undergoing penectomy, and in 69% of those with node-positive disease [11]. In another retrospective analysis of 53 archival PCa specimens, 40% of cases were positive for PDL-1, with 38% of node-positive tumors showing PD-L1 expression [12]. As a result of the biological rationale and the compelling need for additional systemic options, a few ICIs have been explored in patients with advanced PCa, with promising results. Chahoud [13] and others originally reported on two cases who received anti-PD-1 agent pembrolizumab after chemotherapy, radiation and surgery: one man with a high tumor mutation burden achieved a complete response maintained for >38 months and another man achieved a partial response, maintained for >18 months. Conversely, a report of a Phase II trial conducted in various rare malignancies showed that pembrolizumab was not capable of providing any benefits in two patients with microsatellite-stable PCa, while a durable response was obtained in a single man with a microsatellite instability high tumor [14]. Combination of anti-CTLA-4 agent ipilimumab and anti-PD-1 agent nivolumab administered at standard doses was associated with a prominent response in a patient refractory to paclitaxel, ifosfamide and cisplatin who was selected for treatment with ICIs on the grounds of the results of extensive molecular analysis showing high PDL-1 expression, microsatellite instability and tumor mutational burden, as well as alterations in DNA mismatch repair genes [15]. Conversely, in a single-arm, multicohort, Phase II trial, assessing nivolumab and ipilimumab in patients with advanced rare genitourinary cancers not selected on the grounds of any molecular biomarker, only two of five patients evaluable for radiological response showed stable disease [16]. Finally, anti-PD-1 agent cemiplimab tested in patients with advanced squamous cell carcinoma of the skin was associated with a partial response in a single patient with metastatic PCa included in the trial [17], and a complete response was reported in a separate case report of an HIV+ patient receiving cemiplimab [18].

Overall, the scanty data reported suggest that some, albeit unsatisfactory progress has been made over the past 5 years in the field of immunotherapy of PCa [19]. One unexplored, yet potentially effective strategy is the use of combination therapy based on ICIs plus chemotherapy or biological agents. One meta-analysis including quantitative data from 5388 patients with different solid tumors showed that ICI plus chemotherapy was associated with a significantly higher tumor response rate (relative risk: 2.51; 95% CI: 1.82–3.47), decreased chances of progression (hazard ratio [HR]: 0.62; 95% CI: 0.53–0.74), and death (HR: 0.69; 95% CI: 0.61–0.78), compared with single agent ICI or chemotherapy [20]. Considering that anti-PD-1 agents such as pembrolizumab have been successfully combined with cisplatin and fluorouracil [21], combination of cisplatin + 5-fluoruracil, which has proven to be active as a first-line therapy of advanced PCa [5], with pembrolizumab appears to be an appealing experimental therapeutic option. Conversely, in patients who are unfit for chemotherapy, but are candidates for anti-EGFR therapy on the basis of molecular data, combination of an anti-PD-1 agent like nivolumab and an anti-EGFR agent like cetuximab has proven to be well tolerated [22] and may also represent a promising therapeutic option. Finally, with the hurdles associated with the rarity of the disease, which makes it difficult for pharmaceutical companies to obtain regulatory approval, patient selection for ICIs based on known predictive markers, including microsatellite instability, tumor mutational burden, PDL-1 expression, will be mandatory before treatment with an ICI, especially as these biomarkers become more widely available in clinical practice. The rarity of this disease, as in the case of other rare tumors, makes it more difficult to draw an exact line between an experimental and a nonexperimental setting even in common clinical practice. An international effort made by independent scientists, governmental institutions and pharmaceutical companies is compelling in order to increase funding to support research against such a deadly and neglected disease; provide access to nonapproved, high-cost novel agents based on a sound scientific rationale; and increase data sharing among clinicians and researchers through the use of web-based public repositories, specifically designed to collect data on rare diseases. These goals can be better achieved if both patients and clinicians are aware that PCa, as a rare disease, must be treated at referral centers with proven expertise.

## References

[1] Sonpavde G, Pagliaro LC, Buonerba C, Dorff TB, Lee RJ, Di Lorenzo G. Penile cancer: current therapy and future directions. Ann. Oncol. 24(5), 1179–1189 (2013). 23293117 10.1093/annonc/mds635 PMC4047287

[2] Siegel RL, Miller KD, Jemal A. Cancer statistics, 2020. CA Cancer J. Clin. 70(1), 7–30 (2020). 31912902 10.3322/caac.21590

[3] Di Lorenzo G, Federico P, Buonerba C Paclitaxel in pretreated metastatic penile cancer: final results of a Phase II study. Eur. Urol. 60(6), 1280–1284 (2011). 21871710 10.1016/j.eururo.2011.08.028

[4] Pagliaro LC, Williams DL, Daliani D Neoadjuvant paclitaxel, ifosfamide, and cisplatin chemotherapy for metastatic penile cancer: a Phase II study. J. Clin. Oncol. Off. J. Am. Soc. Clin. Oncol. 28(24), 3851–3857 (2010). 10.1200/JCO.2010.29.5477 PMC2940402 20625118

[5] Di Lorenzo G, Buonerba C, Federico P Cisplatin and 5-fluorouracil in inoperable, stage IV squamous cell carcinoma of the penis. BJU Int. 110(11 B), E661–E666 (2012). 22958571 10.1111/j.1464-410X.2012.11453.x

[6] Di Lorenzo G, Buonerba C, Gaudioso G EGFR mutational status in penile cancer. Expert Opin. Ther. Targets 17(5), 501–505 (2013). 23517177 10.1517/14728222.2013.783571

[7] Di Lorenzo G, Perdonà S, Buonerba C Cytosolic phosphorylated EGFR is predictive of recurrence in early stage penile cancer patients: a retropective study. J. Transl. Med. 11(1), (2013). 10.1186/1479-5876-11-161 PMC3717121 23819610

[8] Di Lorenzo G, Buonerba C, Ferro M The epidermal growth factor receptors as biological targets in penile cancer. Expert Opin. Biol. Ther. 5(4), 473–476 (2015). 10.1517/14712598.2015.993377 25496291

[9] Buonerba C, Di Lorenzo G, Pond G Prognostic and predictive factors in patients with advanced penile cancer receiving salvage (2nd or later line) systemic treatment: a retrospective, multi-center study. Front. Pharmacol. 7(DEC), (2016). 10.3389/fphar.2016.00487 PMC5168461 28066240

[10] Sun L, Zhang L, Yu J Clinical efficacy and safety of anti-PD-1/PD-L1 inhibitors for the treatment of advanced or metastatic cancer: a systematic review and meta-analysis. Sci. Rep. 10(1), 2083 (2020). 32034198 10.1038/s41598-020-58674-4 PMC7005709

[11] De Bacco MW, Carvalhal GF, MacGregor B PD-L1 and p16 expression in penile squamous cell carcinoma from an endemic region. Clin. Genitourin. Cancer. 18(3), e254–e259 (2020). 32139302 10.1016/j.clgc.2019.10.014

[12] Cocks M, Taheri D, Ball MW Immune-checkpoint status in penile squamous cell carcinoma: a North American cohort. Hum. Pathol. 59, 55–61 (2017). 27663086 10.1016/j.humpath.2016.09.003

[13] Chahoud J, Skelton WP 4th, Spiess PE Case report: two cases of chemotherapy refractory metastatic penile squamous cell carcinoma with extreme durable response to pembrolizumab. Front. Oncol. 10, 615298 (2020). 33425770 10.3389/fonc.2020.615298 PMC7793656

[14] Hahn AW, Chahoud J, Campbell MT Pembrolizumab for advanced penile cancer: a case series from a Phase II basket trial. Invest. New Drugs (2021). 10.1007/s10637-021-01100-x PMC11948987 33770291

[15] Baweja A, Mar N. Metastatic penile squamous cell carcinoma with dramatic response to combined checkpoint blockade with ipilimumab and nivolumab. J. Oncol. Pharm. Pract. 27(1), 212–215 (2021). 32380900 10.1177/1078155220922602

[16] McGregor BA, Campbell MT, Xie W Results of a multicenter, Phase II study of nivolumab and ipilimumab for patients with advanced rare genitourinary malignancies. Cancer 127(6), 840–849 (2021). 33216356 10.1002/cncr.33328 PMC13213840

[17] Migden MR, Rischin D, Schmults CD PD-1 blockade with cemiplimab in advanced cutaneous squamous-cell carcinoma. N. Engl. J. Med. 379(4), 341–351 (2018). 29863979 10.1056/NEJMoa1805131

[18] Proietti I, Skroza N, Filippi L Pembrolizumab for advanced penile cancer: a case series from a Phase II basket trial. Dermatol. Ther. e14744 (2021). 33770291 10.1007/s10637-021-01100-x PMC11948987

[19] Buonerba C, Pagliuca M, Vitrone FM Immunotherapy for penile cancer. Future Sci. OA3 (3), FSO195 (2017). 10.4155/fsoa-2017-0031 PMC5583688 28883996

[20] Nie R-C, Zhao C-B, Xia X-W The efficacy and safety of PD-1/PD-L1 inhibitors in combination with conventional therapies for advanced solid tumors: a meta-analysis. Biomed Res. Int. 2020, 5059079 (2020). 32461994 10.1155/2020/5059079 PMC7225910

[21] Shitara K, Van Cutsem E, Bang Y-J Efficacy and safety of pembrolizumab or pembrolizumab plus chemotherapy vs chemotherapy alone for patients with first-line, advanced gastric Cancer: The KEYNOTE-062 Phase III randomized clinical trial. JAMA Oncol. 6(10), 1571–1580 (2020). 32880601 10.1001/jamaoncol.2020.3370 PMC7489405

[22] Chung CH, Bonomi MR, Steuer CE Concurrent cetuximab (CTX) and nivolumab (NIVO) in patients with recurrent and/or metastatic (R/M) head and neck squamous cell carcinoma (HNSCC): results of Phase II study. J. Clin. Oncol. 38(Suppl. 15), 6515 (2020).

